# Validation of the SF-36 in patients with endometriosis

**DOI:** 10.1007/s11136-013-0442-5

**Published:** 2013-07-13

**Authors:** Donald E. Stull, Radek Wasiak, Noemi Kreif, Mireia Raluy, Antje Colligs, Christian Seitz, Christoph Gerlinger

**Affiliations:** 1 RTI Health Solutions, Manchester, UK; 2 United BioSource Corporation, 26-28 Hammersmith Grove, London, UK; 3 Bayer Pharma AG, Berlin, Germany; 4 Gynecology, Obstetrics and Reproductive Medicine, University Medical School of Saarland, 66421 Homburg, Saar Germany

**Keywords:** Endometriosis, Health-related quality of life, SF-36, Psychometric validation

## Abstract

**Objectives:**

Endometriosis presents with significant pain as the most common symptom. Generic health measures can allow comparisons across diseases or populations. However, the Medical Outcomes Study Short Form 36 (SF-36) has not been validated for this disease. The goal of this study was to validate the SF-36 (version 2) for endometriosis.

**Methods:**

Using data from two clinical trials (*N* = 252 and 198) of treatment for endometriosis, a full complement of psychometric analyses was performed. Additional instruments included a pain visual analog scale (VAS); a physician-completed questionnaire based on patient interview (modified Biberoglu and Behrman—B&B); clinical global impression of change (CGI-C); and patient satisfaction with treatment.

**Results:**

Bodily pain (BP) and the Physical Component Summary Score (PCS) were correlated with the pain VAS at baseline and over time and the B&B at baseline and end of study. In addition, those who had the greatest change in BP and PCS also reported the greatest change on CGI-C and patient satisfaction with treatment. Other subscales showed smaller, but significant, correlations with change in the pain VAS, CGI-C, and patient satisfaction with treatment.

**Conclusions:**

The SF-36—particularly BP and the PCS—appears to be a valid and responsive measure for endometriosis and its treatment.

## Background

Endometriosis is a common, chronic gynecological disease among women of reproductive age. It is defined by the growth of endometrium-like tissue outside the uterine cavity, including the ovaries and other pelvic structures [1]. The condition is associated with a variety of symptoms, with the main clinical symptoms such as dysmenorrhea (pain on menstruation), dyspareunia (painful intercourse), dyschezia (painful bowel movements), lower back pain, and chronic pelvic pain [1–6]. It has been suggested that chronic pelvic pain is the most important clinical factor of endometriosis [7] and is commonly reported among women with the condition. Moreover, it is a progressive disease that worsens over time [8].

Among gynecological conditions, endometriosis is the third leading cause of gynecological hospitalization in the United States [9]. Exact prevalence is unknown as the endometriosis can only be definitively diagnosed during pelvic surgery, usually laparoscopy or laparotomy; therefore, most prevalence estimates are made on the basis of surgical populations [10]. Estimates vary widely [11], but the disease is generally estimated to occur in 5–10 % of women in the general population [2, 10–15]. In women with pelvic pain, the prevalence is even estimated to be 3 or more times higher [2, 8, 16].

In addition to clinical symptoms, women with endometriosis experience a range of non-clinical symptoms. Depression and isolation are feelings often experienced. Women with endometriosis report worse emotional well-being than women with a primary diagnosis of depression, hypertension, diabetes mellitus, heart disease, and arthritis [17]. Problems with sex life and relationships are also common [17, 18]. Women with endometriosis have reported having less intercourse and more frequent interruption of intercourse due to pain [4]. Additionally, women with endometriosis have difficulty in fulfilling work and social commitments [19] and often report fatigue or lack of energy [6, 20].

The existence of endometriosis-associated symptoms has an adverse impact on physical, mental, and social well-being and therefore a negative effect on health-related quality of life (HRQOL) [19, 21–24]. This impact is additionally magnified by the degree of severity of the condition; more severe cases are associated with greater reduction of HRQOL [18, 25].

Treatments aim to alleviate or significantly reduce pain, thereby reducing the burden of the illness. For chronic pain, the most important measures of treatment response and reduction in illness burden involve patient-reported outcomes (PROs) because the patient is the most important judge of whether changes are important or meaningful [26, 27]. Clinical trials of endometriosis treatment have reported significant improvement in HRQOL assessed using PRO measures following treatment [28–36]. Disease-specific PRO measures have been developed and as measures of treatment efficacy, such as the Endometriosis Health Profile—30 [37]. In addition, generic HRQOL PRO measures are also used in studies of endometriosis, with the Medical Outcomes Study Short Form 36 (SF-36) being one of the most common [22].

Although disease-specific instruments are more sensitive to disease experiences than generic instruments [38], the SF-36 has advantages of allowing comparisons across diseases and between patients’ scores with those of the general public. This information is useful in establishing a thorough understanding of disease impact in relation to other conditions and healthy individuals. The SF-36 has been found to be responsive to change in health status in women receiving treatment for endometriosis [39] but has not been validated specifically for this condition.

The purpose of this study is to evaluate the validity of the SF-36 in endometriosis, using data from two clinical trials. A secondary objective is to examine the responsiveness and minimally important difference (MID) of the SF-36 in patients with endometriosis. Use of the SF-36 in endometriosis offers at least two advantages over disease-specific measures for this condition or its symptoms. First, as a generic health measure, it allows comparisons of HRQOL of women with endometriosis with HRQOL experiences of other diseases. Second, generic health measures tend to be less sensitive to the disease experience than disease-specific measures [38]. Thus, to the extent that the SF-36 detects improvements resulting from treatment, this would be stronger evidence of a treatment effect.

## Methods

### Data

Data came from two phase III studies of a treatment for endometriosis-related symptoms. Study A is a 24-week, multicenter, open-label, randomized, parallel-group, non-inferiority study investigating the efficacy and safety of daily oral administration of 2 mg dienogest versus intramuscular administration of 3.75 mg leuprorelin acetate every 4 weeks for the treatment for symptomatic endometriosis in 248 subjects with endometriosis [40]. Study B is a 12-week, double-blind, randomized, placebo-controlled, parallel-group study designed to investigate the efficacy and safety of daily oral administration of 2 mg dienogest versus placebo for pelvic pain in 198 subjects with endometriosis [41].

### Measures

Data from three PRO measures and two clinician-completed measures were collected in both trials. Two of the PROs and both clinician-completed measures were used to validate the SF-36. The three PROs are described first below followed by the descriptions of the clinician-completed instruments.

#### Medical Outcomes Study Short Form 36

The SF-36 is one of the most widely used generic measures of health [2] and is commonly used in studies of endometriosis and common gynecological conditions, including endometriosis [22]. The SF-36 is a self-administered, generic health status questionnaire that measures 8 health concepts [42, 43]: “physical functioning (PF), role limitations due to physical problems (RP), bodily pain (BP), general health perception (GH), vitality (VT), social functioning (SF), role limitations due to emotional problems (RE), and mental health (MH).” The typical factor structure of the SF-36 hypothesizes that PF, RP, BP, and GH are subscales of the physical component, while RE, VT, MH, and SF are subscales of the mental component.

Scores can be calculated for each domain or by Physical and Mental Component Summary Scores (PCS and MCS) [43]. Scores are generally transformed to a range from 0 to 100 for the 8 subscales; the two components are normed with *z*-scores of mean = 50.0 and SD = 10.0. For all subscales and both components, a higher score indicates better health status on each dimension. In this study, version 2 of SF-36 was used.

#### The pelvic pain visual analog scale

As pain is the most dominant symptom of endometriosis, patients indicated their endometriosis-associated pelvic pain on a 100 mm visual analog scale (VAS). The ends of the VAS were anchored with the descriptions (0) “absence of pain” to (100) “unbearable pain.”

#### Patient satisfaction with treatment

Only patients in Study B rated their satisfaction with treatment (very much satisfied, much satisfied, minimally satisfied, neither satisfied nor dissatisfied, minimally dissatisfied, much dissatisfied, very much dissatisfied). This was used to assess the extent to which changes in the SF-36 subscales and components show differences for varying levels of treatment satisfaction.

#### The Biberoglu and Behrman severity profile

The Biberoglu and Behrman scale (B&B) [44] is a physician-completed questionnaire based on patient interview referring to the previous 4 weeks. The B&B evaluates three cardinal symptoms reported by endometriosis patients: dysmenorrhea, dyspareunia, and pelvic discomfort/pain. Each symptom has four possible intensities (0 = none, 1 = mild, 2 = moderate, and 3 = severe) based on the patient’s self-assessment of pain and the gynecological palpation by the attending physician. A summary score on these three items (0 = none, 1–3 = mild, 4–6 = moderate, and 7–9 = severe) is calculated. Physicians also rate 2 items on the same 0–3 scale that evaluate physical signs of endometriosis: pelvic tenderness and induration, yielding a summary score from 0 (none) to 5–6 (severe). A total symptom severity score is calculated by summing the pain/discomfort and physical signs scales.

#### Clinical global impressions of change

At the end-of-study visit, only in Study B, the investigator assessed each patient’s improvement relative to symptoms at baseline on the clinical global impressions of change (CGI-C) [45], a 7-point scale: 1 = “Very much improved,” 2 = “Much improved,” 3 = “Minimally improved,” 4 = “No change,” 5 = “Minimally worse,” 6 = “Much worse,” 7 = “Very much worse.” CGI-C was administered at week 12 in the placebo-controlled study.

### Assessment points

The SF-36 was completed at baseline and end of study (week 24 for Study A; week 12 for Study B). The pelvic pain VAS was completed at baseline and every 4 weeks in both studies. The B&B was completed at baseline and week 12 for both studies, and week 24 for Study A. Finally, the CGI-C and patient satisfaction with treatment were completed at week 12 for Study B only.

### Analyses

As the factor structure of the SF-36 is generally well established and because sample sizes for the two trials were relatively small, analyses began with confirmatory factor analyses (CFA). A confirmatory factor analysis of the SF-36 was first conducted on Study A at baseline. Once a satisfactory measurement model was obtained, confirmatory analyses were conducted using baseline data from Study B to see whether a comparable factor structure was supported. The remaining psychometric analyses were conducted on both trial datasets separately based on the results of the factor structure from the CFA.

#### Confirmatory factor analysis

Confirmatory factor analyses using structural equation modelling were conducted to confirm the measurement model and fit of subscales within the hypothesized structure of the SF-36. The analyses assessed the fit of an 8-factor and 2-summary-score solution as specified in the SF-36 standard scoring manual [46]. Since confirmatory analyses require relatively large sample sizes with sample size requirements increasing as models become more complex [47], the analyses were performed at the level of the subscales and components, not the items, using total scores for each subscale due to the relatively small sample sizes in each trial (Study A = 252 and Study B = 198). Specifically, the factors of physical functioning, role physical, bodily pain, and general health were hypothesized as subscales of the Physical Component Score and the factors of role emotional, vitality, mental health, and social functioning were hypothesized as subscales of the Mental Health Component Score [46]. Overall model fit was assessed and factor loadings were evaluated for acceptable magnitude (factor loadings of 0.40 are conventionally considered acceptable).

Adequacy of fit was assessed using several fit indices: Comparative Fit Index (CFI), standardized root mean residual (SRMR), and root mean square error of approximation (RMSEA) [47, 48]. In addition, modification indices were examined for any anomalous results (e.g., correlated errors, secondary loadings that were not explicitly modelled).

In the context of structural equation modelling, several fit statistics provide information about the adequacy of the model to explain the data [47]. In general, a model explains the data well if the CFI, that is, the difference between the hypothesized model and a null model, is 0.9 or better, though there is some disagreement about 0.9 or 0.95 as the lower threshold for the CFI [48]. The SRMR measures the mean absolute difference between observed and model-implied correlations; values of <0.1 are considered acceptable [48]. As such, the SRMR is a measure of “badness of fit” as a larger value represents a larger discrepancy between the hypothesized model and the data. Finally, the RMSEA is also a measure of the “badness of fit,” assessing the discrepancy between the predicted and observed data per degree of freedom; values <0.08 are considered acceptable [49]. The 90 % confidence interval (CI) for the RMSEA should be narrow, giving additional confidence in the estimate. Once the model had been run and acceptable fit was achieved using baseline data from Study A, the model was confirmed using baseline data from Study B.

#### Internal consistency reliability

Once the factor structure of the SF-36 was confirmed, internal consistency was assessed (Cronbach’s alpha; standardized items are reported, though the results for unstandardized items were identical to the third decimal place) for each subscale first using baseline data from Study A and then with baseline data from Study B.

Test–retest reliability was not performed due to the relatively long lags between SF-36 assessments (Study A: 24 weeks; Study B 12 weeks).

The internal consistency reliability was assessed using Cronbach’s formula for coefficient alpha:

$$\alpha = \frac{{N \cdot \bar{c}}}{{\left( {\bar{\upsilon } + \left( {N - 1} \right) \cdot \bar{c}} \right)}}$$

where *N* is the number of components (items or tests), $\bar{\upsilon }$ equals the average variance, and $\bar{c}$ is the average of all covariances between the components. In addition, the item-rest correlation (i.e., the multiple correlation coefficient “R” for each item, having regressed each item on the remaining items in the scale) was examined to see whether any items are less correlated with the remaining items.

The standardized alpha was presented. This was based on standardized scores (mean = 0 and standard deviation = 1) for each of the items. There are no tests of statistical significance for alpha; the values are presented descriptively on an interval level scale from 0 to 1.0, with higher scores indicating a more reliable (precise) instrument. The target Cronbach’s standardized alpha is at least 0.70, though patterns of item-to-item correlations and item-to-total correlations are also important, as are the number of items in the subscale. Moreover, an alpha that is too high (e.g., approaching 1.0) can indicate a set of items that are likely to be redundant, so this is not optimal.

#### Construct validity

Construct validity, the extent to which the instrument measures what it is intended to measure, was evaluated in a variety of ways. Specifically, SF-36 subscale and component scores were correlated with the pelvic pain VAS item (at baseline and end of study for both studies), B&B (pelvic discomfort and pain and total score; at baseline and end of study for both studies), and patient treatment satisfaction rating (at week 12 in Study B). Spearman correlation coefficients were used to evaluate these relationships.

#### Known groups/discriminant validity

Known groups/discriminant validity was assessed through the ability of the SF-36 subscale and component scores to discriminate between groups of patients according to the levels of symptom severity, based on the B&B symptom severity using analysis of variance (ANOVA) with Scheffe’s post hoc comparisons. Mean differences between four symptom severity groups at baseline were compared to assess the relationship between SF-36 scores and symptom severity item scores at baseline for both studies. Subjects were stratified depending on their symptom severity item scores. The groups were 0 (none), 1 (mild), 2 (moderate), and 3 (severe).

A similar ANOVA strategy evaluated differences in mean SF-36 subscale and component scores by VAS pain severity groups. Quartiles of VAS pain severity groups were created after examination of descriptive statistics, and Scheffe’s post hoc comparisons of mean SF-36 scores between quartiles were carried out.

Finally, for Study B, the mean change in SF-36 was compared for different values of the CGI-C. These ANOVAs indicate whether those for whom the clinician rated as “Very much improved” had significantly higher mean scores on the SF-36 subscales and components than those with clinician ratings of change that were less improved.

#### Responsiveness and minimal important difference

To evaluate responsiveness of the SF-36 subscale and component scores, correlations were computed between changes in the SF-36 and changes in the pain VAS for Study A, and between changes in the SF-36 with changes in the pain VAS and the CGI-C for Study B.

Two methods—a priori and data-based—were used to establish change thresholds for assessing the relationship between minimal change in pain and the corresponding change in the SF-36 bodily pain subscale and the PCS. First, we used as a priori thresholds those suggested by Farrar et al. [50] to anchor important changes in pain using a 0–10 numerical rating scale. Farrar et al. [50] found that changes of 1–2 points were considered small but important to patients. Applying this finding to the 0–100 (“absence of pain” to “unbearable pain”) VAS scale, those with a 10- to 29-point change toward the “0” end on the VAS scale were considered as having a small but important change between baseline and end of study, while VAS reductions of 30 points or more were considered moderate to large improvements. Therefore, VAS improvements of 10–29 points represent a “responder,” and changes in the VAS of less than 10 points in either direction (i.e., ±9 points) were considered the stable group (“non-responder”).

Changes in VAS scores were grouped into 5 change categories:

- Decrease of at least 30 mm (very much improved)
- Decrease between 10 and 29 mm (minimally improved)
- Decrease of 9 mm up to an increase of 9 mm (no change)
- Increase between 10 and 29 mm (worse)
- Increase of at least 30 mm (very much worse).

The second approach used the distributions of change based on the data in each study to establish change thresholds rather than using a priori thresholds, that is, based on the histograms of the change scores in the pain VAS, and categories of “minimal change” and “no change” were established. Interestingly, the category of “minimal change” was consistent with that noted above: a change of 10–30 points, while the “no change” group had a slightly larger range (−10–10).

A step-wise triangulation approach was used to establish an MID for the SF-36 subscales. First, distribution-based approaches were used to evaluate MID for Study A and then for Study B. An anchor-based method using the CGI-C measure from Study B was used to confirm an MID. Another way of exploring the MID is to use receiver operating characteristic (ROC) curves to look at sensitivity and specificity for different cut points when comparing patients who improve versus those who show no change on the SF-36 over the trial. The final cut point is one that strikes a balance between sensitivity and specificity, and correctly identifies the greatest proportion of patients with detectable improvement without incorrectly identifying patients as having improvement when in fact they did not. Two different ROC curves were computed based on the pain VAS categories of change noted above. In Farrar et al. [50], a priori category of “minimally improved” was compared with that of “no change.” In a second analysis, the data-derived categories of “minimally improved” and “no change” were compared.

## Results

Table 1 presents the baseline patient characteristics for Studies A and B.

**Table 1:** Patient characteristics at baseline

	Study A (*N* = 252)	Study B (*N* = 198)
Age mean (SD)	30. 8 (5.9)	31.4 (6.4)
Race/ethnicity *n* (%)		
Caucasian	247 (98.0 %)	196 (99.0 %)
Hispanic	1 (0.4 %)	
Asian	3 (1.2 %)	2 (1.0 %)
Other	1 (0.4 %)	
Country of origin *n* (%)		
Germany	166 (65.9 %)	60 (30.3 %)
Italy	20 (7.9 %)	19 (9.6 %)
Austria	10 (4.0 %)	
Poland	36 (14.3 %)	
Portugal	1 (0.4 %)	
Spain	19 (7.5 %)	
Ukraine		119 (60.1 %)

Baseline SF-36	*n* (%) at floor	*n* (%) at ceiling	*n* (%) at floor	*n* (%) at ceiling
Physical functioning	0	54 (21.4)	0	21 (10.7)
Role physical	65 (25.9)	84 (33.5)	48 (24.4)	48 (24.4)
Bodily pain	14 (5.6)	11 (4.4)	2 (1.0)	3 (1.5)
General health perceptions	0	2 (0.8)	1 (0.5)	0
Vitality	1 (0.4)	0	2 (1.0)	0
Social functioning	2 (0.8)	61 (24.2)	1 (0.5)	22 (11.2)
Role emotional	52 (20.6)	125 (49.6)	44 (22.3)	83 (42.1)
Mental health	0	0	0	1 (0.5)

Pain VAS	Mean	SD	Mean	SD
Overall	54.4	26.6	56.9	17.9
Quartile 1	17.8	–	34.9	–
Quartile 2	46.5	–	48.3	–
Quartile 3	65.4	–	62.8	–
Quartile 4	86.6	–	80.6	–

B&B pelvic pain severity	*n*	%	*n*	%
None	2	0.8	1	0.5
Mild	69	27.4	34	18
Moderate	141	56	130	68.8
Severe	40	15.9	24	12.7

### Confirmatory factor analysis

**Fig. 1:**
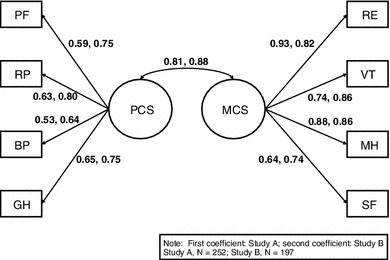
Confirmatory factor analysis factor loadings (standardized)

The model fit statistics of the CFA for both trials are presented in Table 2. The factor loadings for both trials and correlations between the PCS and MCS are presented in Fig. 1. The CFI was 0.92 and 0.91 for Studies A and B, respectively, between the recommended thresholds of 0.9 and 0.95. The SRMR was below the threshold deemed acceptable for both of the studies, further confirming the hypothesized factor structure, that is, the mean differences between the data-derived correlations and those implied by the model were trivial. However, the reported RMSEA values were outside of the acceptable range, especially for Study B where the 90 % CI was entirely above the recommended threshold of 0.08. It is possible, however, for the RMSEA to be unacceptably high in simpler models, such as those analyzed here [51]. In this case, both the CFI and SRMR indicate acceptable fit and the RMSEA can be ignored. Also, as shown in Fig. 1, all factor loadings were above an acceptable threshold of 0.40.

**Table 2:** Confirmatory factor analysis model fit statistics

	Study A (*N* = 252)	Study B (*N* = 197)
Chi-square (*df*)	64.97 (19)	107.15 (19)
Comparative Fit Index	0.92	0.91
Root mean square error of approximation	0.1	0.15
90 % CI for root mean square error of approximation	0.07–0.13	0.13–0.18
Standardized root mean residual	0.05	0.05

*CI* confidence interval, *df* degrees of freedom

### Internal consistency reliability

The results of this part of the analysis are presented in Table 3. Although the confirmatory factor analyses needed to be performed with the subscales components, the internal consistency reliabilities could be calculated for the items within each subscale. In general, internal consistency reliability of the subscales was acceptable with alpha above the generally acceptable reliability value of 0.70. The two scales that were closest to this threshold were general health for Study A (alpha = 0.73) and role physical for Study B (alpha = 0.75). The “alpha-if-deleted” changed little for each of the eight subscales suggesting a high degree of internal consistency for each subscale. The one notable exception was item 5b (“Accomplished less than you would like”) for role emotional. This was the case for both trials. Standardized and unstandardized values were calculated, but were negligibly different (at the third decimal place).

**Table 3:** Reliabilities (Cronbach’s alpha) of SF-36 subscales and components

	Study A (*N* = 252)	Study B (*N* = 198)
	Scale Alpha	Scale Alpha
SF-36 subscales and items		
Physical functioning	0.88	0.87
Role physical	0.83	0.75
Bodily pain	0.79	0.81
General health	0.73	0.81
Vitality	0.82	0.82
Social functioning	0.80	0.83
Role emotional	0.81	0.76
Mental health	0.87	0.85
SF-36 components and subscales		
Physical health component	0.89	0.92
Mental health component	0.92	0.93

### Construct validity

Construct validity was assessed by correlations between the SF-36 subscales and components and the pain VAS and B&B pelvic discomfort and pain scores. Results of construct validity analyses with the pain VAS for both trials are presented in Table 4. In Study A, five SF-36 subscales (PF, RP, BP, VT, and MH) and one component (PCS) were statistically significantly correlated with the pain VAS at baseline. At end of study, all subscales and both components were statistically significantly related to the pain VAS. For Study B, a similar, though slightly more compelling, set of results emerged. Both SF-36 components and all subscales, except GH, were statistically significantly related to the pain VAS at baseline. At end of study, like Study A, all subscales and both components were statistically significantly related to the pain VAS, though the correlations for Study B were generally larger except for MH. Of particular note is that the correlation of BP with the pain VAS was moderate [52] for both studies at baseline and end of study. The PCS was weakly correlated with the pain VAS for Study A and Study B at baseline; at end of study, it was moderately correlated in both studies (*r* = −.41 and −.44). Other dimensions show only a weak or sometimes very weak relationship.

**Table 4:** Pearson correlations between SF-36 subscale and component scores and pain VAS, baseline and end of study

	Study A		Study B	
	*N*	*r*	*N*	*r*
Baseline SF-36				
Physical functioning	245	−.27^‡^	197	−.20^†^
Role physical	244	−.24^‡^	197	−.28^‡^
Bodily pain	245	−.48^‡^	197	−.47^‡^
General health	242	−.10	197	−.05
Vitality	245	−.20^†^	197	−.43^‡^
Social functioning	245	−.11	197	−.27^‡^
Role emotional	245	−.08	197	−.24^‡^
Mental health	245	−.14*	197	−.17*
Physical health component score	241	−.38^‡^	197	−.29^‡^
Mental ealth component score	241	−.06	197	−.24^‡^
EOS SF-36				
Physical functioning	221	−.26^‡^	189	−.31^‡^
Role physical	220	−.32^‡^	189	−.32^‡^
Bodily pain	221	−.57^‡^	189	−.63^‡^
General health	218	−.21^†^	188	−.28^‡^
Vitality	221	−.31^‡^	189	−.42^‡^
Social functioning	221	−.20^†^	189	−.32^‡^
Role emotional	218	−.24^‡^	187	−.34^‡^
Mental health	221	−.32^‡^	189	−.28^‡^
Physical health component score	214	−.41^‡^	186	−.44^‡^
Mental health component score	214	−.23^‡^	186	−.31^‡^

* *P* ≤ 0.05; ^†^ *P* ≤ 0.01; ^‡^ *P* ≤ 0.001

Spearman correlations between SF-36 subscales and components and the B&B pelvic discomfort and pain exhibited a similar pattern of correlations at baseline and end of study for both studies (results not shown). Correlations tended to be larger at end of study than at baseline and for Study B compared with Study A. Not surprisingly, BP had the strongest correlation of the subscales with the B&B pelvic discomfort and pain; the PCS had a slightly weaker correlation with the B&B pelvic discomfort and pain.

### Known groups/discriminant validity

Mean differences in SF-36 subscale and component scores were compared by level of symptom severity on the B&B symptom severity (none, mild, moderate, severe) using ANOVA with Scheffe’s post hoc comparisons. Results of these analyses are presented in Table 5 for baseline and end of study for both studies (for details by B&B symptom severity, see Appendix Table 9). For Study A at baseline, with the exception of RE and the MCS, all SF-36 subscales and the PCS were significantly associated with levels of symptom severity. At end of study for Study A, both SF-36 components and all subscales, except GH, MH, and SF, and the MCS were significantly associated with levels of symptom severity when comparing pelvic pain severity groups: patients with lower B&B symptom severity scores (i.e., less severe) had better mean SF-36 subscale and component scores. The association was particularly strong for the bodily pain SF-36 score and the PCS.

**Table 5:** Discriminant validity of the SF-36 scores: ANOVA by Biberoglu & Behrman symptom severity level at baseline and end of study

	Baseline		End of study	
	B&B severity—Study A	B&B severity—Study B	B&B severity—Study A	B&B severity—Study B
	*F*	*F*	*F*	*F*
Physical functioning	4.37^†^	2.98*	6.58^†^	7.27^‡^
Role physical	7.25^‡^	3.11*	4.83^†^	7.40^‡^
Bodily pain	14.93^‡^	15.77^‡^	27.98^‡^	27.30^‡^
General health	5.78^†^	2.24	1.15	15.30^‡^
Vitality	5.24^†^	3.84*	4.36*	11.19^‡^
Social functioning	4.19^†^	2.70*	0.83	7.11^‡^
Role emotional	1.21	0.65	3.47*	3.59*
Mental health	2.63*	2.34	2.81	7.59^‡^
Physical health component	11.53^‡^	7.42^‡^	13.57^‡^	18.21^‡^
Mental health component	1.59	1.12	1.52	5.31^‡^

* *P* ≤ 0.05; ^†^ *P* ≤ 0.01; ^‡^ *P* ≤ 0.001

Similar, though somewhat less robust, results were seen for Study B at baseline. Mean scores on PF, RP, BP, VT, SF, and the PCS varied significantly by symptom severity level of the B&B. At end of study, however, mean scores for every SF-36 subscale and component varied significantly by B&B severity level.

### Responsiveness and minimally important difference of the SF-36

Responsiveness of the SF-36 subscales and components was evaluated by examining relationships between changes in the SF-36 and changes in the pain VAS and, for Study B, categories of CGI-C and patient satisfaction with treatment. The scoring on the SF-36 change variables is such that a lower or negative score indicates that the respondent got worse (i.e., their end-of-study score was lower/worse than their baseline score). Conversely, for the change in the pain VAS score, a lower or negative score represents an improvement (i.e., their end-of-study score was lower/better than their baseline score). Table 6 presents correlations between changes in SF-36 subscales and components and changes in the pain VAS and summaries of ANOVA *F* tests for comparisons of the mean changes in SF-36 with categorical changes in the pain VAS (very much improved, improved, no change, worse, very much worse). It was hypothesized that those who reported improvement in pain should also report improvements in their SF-36 scores, especially the BP score.

**Table 6:** ANOVAs assessing mean change in SF-36 by mean change in pelvic pain VAS from baseline to end of study and correlations between changes in SF-36 scores and changes in pain VAS from baseline to end of study

	Correlation of change in SF-36 with change in pain VAS		Change in pain VAS^a^	Change in pain VAS^a^	CGI-C	Patient satisfaction with treatment
	Study A	Study B	Study A	Study B	Study B	
	*r*	*r*	*F-*statistic	*F-*statistic	*F-*statistic	*F-*statistic
∆ Physical functioning	−.26^§^	−.19^‡^	4.59^§^	2.43^†^	1.75	0.65
∆ Role physical	−.24^§^	−.26^§^	5.06^§^	3.26^†^	2.07	4.06^‡^
∆ Bodily pain	−.43^§^	−.62^§^	10.46^§^	21.52^§^	18.40^§^	8.52^§^
∆ General health	−.09	−.21^‡^	2.02	3.14^†^	2.41^†^	2.36^†^
∆ Vitality	−.15^†^	−.30^§^	3.03^†^	4.23^‡^	2.84^†^	2.09
∆ Social functioning	−.03	−.23^‡^	0.81	2.95^†^	1.610	1.67
∆ Role emotional	−.13	−.24^§^	2.83^†^	3.14^†^	2.94^†^	1.84
∆ Mental health	−.07	−.14	1.93	0.73	1.07	0.71
∆ Physical health component	−.37^§^	−.45^§^	8.57^§^	10.35^§^	6.79^§^	6.77^§^
∆ Mental health component	−.02	−.17^†^	1.60	1.40	1.13	1.44

*r* Denotes a correlation coefficient

^†^ *P* ≤ 0.05; ^‡^ *P* ≤ 0.01; ^§^ *P* ≤ 0.001

^a^Very much improved (*n*: Study A = 136; Study B = 60); improved (*n*: Study A = 46; Study B = 78), no change (*n*: Study A = 25; Study B = 45), worse (*n*: Study A = 8; Study B = 3), very much worse (*n*: Study A = 1; Study B = 1)

For both trials, correlations between changes in SF-36 and changes in the pain VAS indicated that decrements in pain VAS scores (i.e., lessening pain) were correlated with improvements in SF-36 subscale and component scores (i.e., greater SF-36 scores). This was particularly notable for the BP subscale and the PCS. These results are reflected in the negative correlations seen in the first two columns of Table 6.

For Study A, those whose mean pain VAS scores improved from baseline to end of study had significantly higher mean improvement in the PCS and all SF-36 subscales, except for GH, MH, and SF. Bodily pain and PCS exhibited a particularly strong and statistically significant relationship. For Study B, those whose mean pain VAS scores improved from baseline to end of study had significantly higher mean improvement in the PCS and all SF-36 subscales, except for MH and MCS.

Improvement based on the CGI-C and patient satisfaction with treatment in Study B was associated with improvement in the SF-36 for several subscales and the PCS. Specifically for the CGI-C, the SF-36 subscales of BP, GH, RE, and VT all had significantly higher means for patients whose clinicians indicated that they had greater improvement in their symptoms since baseline. For patient satisfaction with treatment, mean scores for RP, BP, GH, and PCS were greater for those who had greater satisfaction with treatment for their condition.

Minimally Important Differences analyses

## Study A

Table 7 presents the results of the MID analyses. The results suggest some highly varied MIDs for the SF-36 subscales and components, ranging from about 4 to over 20 for Study A and from under 4 to 20 for Study B. Given the central role that pain plays in endometriosis, the BP subscale and the PCS (of which BP is a component) will be the focus of detailed results. As seen in Table 7, half of the standard deviation of the change in BP is 15. This is slightly larger than the standard error of mean (SEM) (10.4). The SEM describes the error associated with the measure. Wyrwich has shown that this approach closely mirrors results using an approach based on patient global assessment of change [2, 38]. Moreover, these are associated with a substantial effect size (ES) of 1.43, suggesting that a change of this size is meaningful.

**Table 7:** Results of minimally important difference

SF-36 score	*N*	Baseline mean	End of study mean	End of study—baseline	SD, baseline	SD of change	Half SD of change	*α*	SEM^a^	SRM^b^	ES^c^
Study A											
Physical functioning	234	81.8	90.7	8.9	18.4	18.9	9.5	0.88	6.37	0.47	0.49
Role physical	232	54.2	81.1	26.9	40.5	45.2	22.6	0.83	16.70	0.60	0.67
Bodily pain	234	42.4	75.8	33.4	23.3	30.1	15.1	0.80	10.44	1.11	1.43
General health perceptions	228	59.7	62.9	3.2	21.3	19.9	9.9	0.73	11.05	0.16	0.15
Vitality	234	49.1	57.2	8.2	19.3	20.1	10.1	0.83	7.97	0.41	0.42
Social functioning	234	70.2	79.3	9.1	23.5	26.0	13.0	0.80	10.52	0.35	0.39
Role emotional	231	64.6	77.5	12.8	40.4	46.1	23.1	0.82	17.12	0.28	0.32
Mental health	234	60.8	66.2	5.4	19.7	18.3	9.1	0.87	7.12	0.29	0.27
Physical health component	223	43.2	51.4	8.2	8.5	9.3	4.6	0.70	4.64	0.88	0.97
Mental health component	223	43.9	45.7	1.7	11.6	11.6	5.8	0.85	4.50	0.15	0.15
Study B											
Physical functioning	190	76.9	85.2	8.3	18.8	14.6	7.3	0.87	6.79	0.56	0.44
Role physical	190	51.2	70.4	19.2	37.8	36.7	18.4	0.75	18.91	0.52	0.51
Bodily pain	190	42.8	59.1	16.3	16.5	22.4	11.2	0.81	7.19	0.73	0.99
General health perceptions	189	46.1	53.3	7.2	20.7	15.3	7.6	0.81	9.02	0.47	0.35
Vitality	190	49.5	55.3	5.8	19.1	15.5	7.8	0.82	8.10	0.37	0.30
Social functioning	190	64.4	73.2	8.8	23.0	19.9	9.9	0.83	9.49	0.44	0.38
Role emotional	188	59.2	73.4	14.2	40.2	40.5	20.2	0.76	19.72	0.35	0.35
Mental health	190	58.0	62.6	4.5	18.5	14.8	7.4	0.85	7.15	0.31	0.25
Physical health component	187	41.1	46.3	5.3	7.4	6.9	3.5	0.82	3.14	0.76	0.71
Mental health component	187	42.3	44.8	2.5	10.9	9.5	4.7	0.89	3.61	0.26	0.23

*α* = Cronbach’s coefficient of internal consistency reliability; SEM = standard error of measurement; SRM = standardized response mean; ES = effect size

^a^SEM = SD √(1 − *α*)

^b^SRM = change score/SD of the change score

^c^ES = change score/SD at baseline

Receiver operating characteristic curves were calculated to compare those who showed minimal change on the pain VAS versus those who did not change, using the cut points adapted from Farrar et al. [50] (see Table 8). The results of the ROC curves (not presented) suggest that a score between 16 and 21 represents a balance between sensitivity and specificity, correctly classifying 73 % of cases. The second method of setting thresholds of change (using distributions of change based on the data rather than the a priori thresholds suggested by Farrar et al. [50]) suggested that a score of 21 represents a balance between sensitivity and specificity, correctly classifying 70 % of cases (detailed results not presented).

For the PCS, half of the standard deviation of change is 4.6 which is also the value for the SEM (see Table 8). This corresponds to a large ES of 0.97. The score from the ROC curves (using the Farrar et al. [50] method) that balances sensitivity and specificity is 3.7 and correctly classifies 61 % of cases. The score from the ROC curves (using the alternative method for establishing change categories) that balances sensitivity and specificity is 3.8 and correctly classifies 61 % of cases (results not presented).

**Table 8:** Summary of results from minimally important difference analyses

	Bodily pain subscale	
	Study A(*n* = 234)	Study B(*n* = 190)
Half of the standard deviation of change	15	11
Standard error of measurement	10.4	7.2
Effect size of change	1.43	0.99
ROC curves (Farrar et al. method)	16–21^a^	10^b^
ROC curves (alternate method)	21	9
Anchor-based—CGI-C	–	10.7^c^

	Physical Component Summary Score	
	Study A(*n* = 232)	Study B(*n* = 187)
Half of the standard deviation of change	4.6	3.5
Standard error of measurement	4.6	3.1
Effect size of change	0.97	0.71
ROC curves (Farrar et al. method)	3.7^a^	2.9^b^
ROC curves (alternate method)	3.8	3
Anchor-based—CGI-C	–	4.1^c^

CGI-C = clinical global impression of change; ROC = receiver operating characteristic

^a^ *n* = 121

^b^ *n* = 69

^c^ *n* = 109

## Study B

For BP, we see that half of the standard deviation of the change is 11 (see Table 8). This is slightly larger than the SEM (7.2) but these correspond to an ES of 0.99. The results of the ROC curves based on the pre-defined cut points suggested by Farrar et al. [50] suggest that a score of 10 represents a balance between sensitivity and specificity, correctly classifying 63 % of cases (results not presented). Using pain VAS cut points based on the data in the study (alternative method), a score of 9 represents a balance between sensitivity and specificity, correctly classifying 63 % of cases (results not tabled).

For the PCS, half of the standard deviation of change is 3.5 while the value for the SEM is 3.1. This corresponds to an effect size of 0.71. The score from the ROC curves (Farrar et al. [50] method) that balances sensitivity and specificity is 2.9 and correctly classifies 61 % of cases (results not presented). ROC curves using the alternative method for establishing thresholds of change suggest that a score of 3 balances sensitivity and specificity and correctly classifies 61 % of cases (results not tabled).

Using the anchor-based approach (CGI-C) for Study B, comparing “minimally improved” with “no change” in their condition, this corresponded to a BP change of 10.7 and a mean improvement in PCS of 4 (see Table 8).

## Summary of MID results

A summary of the results from the MID analyses is presented in Table 8. The results suggest some triangulation on an MID for both the BP subscale and the PCS, although there was more variability in a possible MID for bodily pain for Study A. For example, a possible MID ranged from 10.4 (SEM) to 21 (ROC curves). A score of around 15–16 seems to fall in the middle of this range for a minimally important change from a patient’s perspective for Study A. For Study B, there was much more consistency in the possible MID values for bodily pain. A score of 11 is a likely value for a minimally important change from the patients’ perspective, based on the half standard deviation of the change, the SEM. The ROC curves suggest a score of 9–10, which is close to the value suggested by the other approaches. Thus, based on these two studies, it appears that a change in the bodily pain subscale between 11 and 16 represents a meaningful change to patients.

Results for the PCS are a little tighter and generally more consistent across the two trials than for the bodily pain subscale. A possible MID ranged from 2.9–3.0 (ROC curves) to 4.6 (half standard deviation of change and SEM). The ROC curves for Study A yielded a value of 3.7–3.8; half standard deviation of change for Study B resulted in a value of 3.5; the anchor-based results using the CGI-C resulted in a value of 4.1. Therefore, it is likely that a change in the PCS in the range of 3.7–3.8 is a meaningful change to patients.

## Discussion

The purpose of this study was to establish the psychometric validity and responsiveness of the SF-36 in endometriosis. A secondary goal was to determine the MID for SF-36 subscales and components. Establishing the psychometric properties and an initial MID for SF-36 is an important step in evaluating the effect of endometriosis on women’s HRQOL and the efficacy of treatments for this condition. That the results from two different trials—an active comparator trial and a placebo-controlled trial—were very similar lends confidence in the results and the robustness of conclusions.

The overall results of the psychometric analyses provide evidence of the validity of the SF-36 for this patient population. The factor structure, construct validity, internal consistency reliability, known groups/discriminant validity, and responsiveness indicate that the SF-36, especially the BP subscale and the PCS, is a valid, reliable, and responsive instrument for measuring HRQOL for women with endometriosis.

To establish the psychometrics of the SF-36, two measures that are generally accepted as appropriate indicators of HRQOL for women with endometriosis—pain VAS and the B&B—were used as comparator measures. Although correlations between the SF-36 and the pain VAS were somewhat mixed (some weak but significant while others were moderate), it performed in expected ways. Further, the results of the ANOVAs with the B&B were consistent with those of the correlations with the pain VAS. Women who reported more pain at baseline on the pain VAS and whose B&B scores were more severe were significantly more likely to have poorer scores on most of the SF-36 subscales, especially the BP and PCS.

Results were also favorable for the SF-36 as a measure that is responsive to change: Patients whose pain VAS scores improved also had improved mean SF-36 scores. Further, those whose pain VAS scores improved the most had the largest improvements in SF-36 scores.

Minimally important difference estimates from this study suggest that, based on the effect size, the BP subscale and the PCS are the two dimensions of the SF-36 that show a strong effect, supporting their ability to detect treatment effects or differences. MID estimates for the bodily pain subscale are in line with those of the developer [53]. For the PCS, MID estimates were close to those that have been published elsewhere, although these were in different indications [54, 55].

The consistency of results across two different trials—active comparator and placebo-controlled—demonstrated that the SF-36 has value in describing the experience of women with endometriosis. This instrument appears to be sensitive to changes in pain or discomfort and differences in effects of treatment. Not surprisingly, given that pain is the most prevalent symptom in endometriosis, BP and PCS, which includes the BP subscale, were especially sensitive to differences in experience and changes in condition.

Recently, using some of the same clinical trial data, Gerlinger and colleagues [56] reported that the minimal important difference (MID) of the pain VAS was 10 mm. This represents the lower threshold used in the present study based on Farrar et al. [50] Thus, the MID values for the SF-36 reported here based on the Farrar et al. approach are likely to be similar to those if the Gerlinger et al. MID value was used.

No single method of establishing an MID is ideal or accepted and each one makes certain assumptions about change [57]. Consequently, researchers use multiple methods and triangulate on a value that is consistent or within a consistent range across the methods used. That was the case in the present study. As seen in Tables 7 and 8, there was general consistency in MID values across the two studies. Thus, while some may take issue with the use of the pain VAS as an anchor and the particular categorizing of the pain VAS, the results from using that anchor correspond reasonably well with the MID results from the other methods used, especially for Study B.

Although there is some debate about the factor structure of the SF-36, there is general consistency in the second-order factor structure (i.e., the subscales that load under the PCS and MCS; [58–60]). The results of the present study are in line with these findings.

That the SF-36, a generic measure of health, appears to be a valid measure for endometriosis and its treatment is advantageous in at least two ways. First, comparisons can be made with other diseases and with general populations, particularly since the PCS has been normed for many populations and diseases. Second, as a generic measure of health, it is likely to be less sensitive to condition-specific changes. The present findings indicate that the SF-36 can detect differences in patients’ conditions and changes in their conditions. Therefore, this suggests that changes in the SF-36 in the context of a clinical trial on the order of the MID reported here are likely to be meaningful and real. This lends confidence in the SF-36 being a valid and responsive measure for endometriosis, and provides evidence that BP and the PCS are especially informative when evaluating the HRQOL impact on patients with diagnosed or suspected endometriosis.

## Appendix

See Table 9.

**Table 9:** Mean SF-36 Domain and Component Scores by pelvic pain symptom severity, baseline, and follow-up

Symptom severity groups	Study A		Study B	
	Baseline	Follow-up	Baseline	Follow-up
	SF-36 ScoreMean (SD) [*n*]	SF-36 ScoreMean (SD) [*n*]	SF-36 ScoreMean (SD) [*n*]	SF-36 ScoreMean (SD) [*n*]
Physical functioning	*	*	*	*
None	91.3 (10.1) [23]	92.8 (13.2) [148]	97.5 (3.5) [2]	93.7 (13.1) [27]
Mild	82.6 (15.6) [84]	87.7 (18.0) [71]	82.5 (14.4) [43]	87.4 (11.9) [99]
Moderate	80.8 (20.0) [113]	84.9 (13.1) [15]	76.4 (18.7) [121]	79.2 (18.4) [59]
Severe	75.6 (22.0) [32]	100.0 (–) [1]	70.6 (22.5) [31]	66.7 (12.1) [6]
Role physical	*	*	*	*
None	72.8 (38.4) [23]	85.4 (27.4) [147]	75.0 (0.0) [2]	92.6 (13.1) [27]
Mild	59.8 (39.4) [84]	76.8 (35.4) [71]	64.5 (32.4) [43]	74.3 (30.9) [99]
Moderate	51.6 (39.3) [112]	61.7 (38.8) [15]	48.5 (39.3) [121]	57.6 (37.5) [59]
Severe	32.8 (39.4) [32]	100.0 (–) [1]	43.5 (35.3) [31]	33.3 (30.3) [6]
Bodily pain	*			*
None	45.7 (30.2) [23]	82.2 (22.6) [147]	71.0 (41.0) [2]	79.6 (21.3) [27]
Mild	51.5 (23.2) [84]	69.6 (19.6) [71]	53.0 (15.2) [43]	62.7 (17.4) [99]
Moderate	38.7 (20.3) [113]	43.1 (24.2) [15]	42.5 (14.9) [121]	46.6 (17.1) [59]
Severe	25.2 (17.6) [32]	42.0 (–) [1]	29.1 (13.6) [31]	30.0 (9.4) [6]
General health	*			*
None	66.7 (18.2) [23]	64.8 (22.6) [146]	67.0 (7.1) [2]	73.4 (19.1) [26]
Mild	62.5 (20.0) [82]	59.3 (20.4) [70]	48.9 (19.8) [43]	54.7 (16.8) [99]
Moderate	58.5 (20.9) [112]	56.9 (19.7) [15]	45.2 (19.5) [121]	44.1 (17.3) [59]
Severe	47.7 (23.7) [32]	72.0 (–) [1]	44.8 (25.7) [31]	34.0 (17.8) [6]
Vitality	*	*	*	*
None	55.9 (19.1) [23]	60.2 (19.5) [148]	67.5 (10.6) [2]	65.0 (17.3) [27]
Mild	51.1 (16.4) [84]	53.2 (19.9) [71]	54.4 (14.3) [43]	58.5 (15.3) [99]
Moderate	47.7 (19.9) [113]	44.0 (20.9) [15]	49.6 (19.0) [121]	47.3 (18.2) [59]
Severe	39.8 (21.5) [32]	60.0 (–) [1]	39.8 (22.2) [31]	35.0 (24.5) [6]
Social functioning			*	*
None	70.7 (21.2) [23]	80.9 (22.4) [148]	81.3 (8.8) [2]	87.0 (15.7) [27]
Mild	74.4 (22.3) [84]	76.4 (22.0) [71]	71.5 (21.9) [43]	76.1 (19.9) [99]
Moderate	69.5 (25.0) [113]	75.8 (26.1) [15]	63.2 (21.7) [121]	65.3 (20.4) [59]
Severe	56.6 (26.6) [32]	100.0 (–) [1]	56.9 (26.6) [31]	41.7 (18.8) [6]
Role emotional				*
None	71.0 (35.3) [23]	81.8 (33.1) [145]	33.3 (47.1) [2]	91.4 (23.7) [27]
Mild	63.9 (42.4) [84]	70.0 (39.9) [71]	69.8 (37.7) [43]	75.9 (31.1) [97]
Moderate	65.8 (40.6) [113]	71.1 (39.6) [15]	56.7 (39.8) [121]	65.5 (38.6) [59]
Severe	52.1 (38.7) [32]	100.0 (–) [1]	54.8 (45.2) [31]	27.8 (44.3) [6]
Mental health	*	*		*
None	51.7 (7.2) [23]	53.4 (7.0) [148]	62.0 (19.8) [2]	74.1 (18.0) [27]
Mild	51.1 (6.5) [84]	50.7 (6.4) [71]	63.0 (18.0) [43]	63.5 (14.5) [99]
Moderate	51.2 (6.8) [113]	49.9 (5.8) [15]	57.6 (17.8) [121]	58.2 (16.6) [59]
Severe	50.0 (8.0) [32]	64.0 (–) [1]	51.0 (20.4) [31]	41.3 (15.9) [6]
Physical health component	*	*	*	*
None	49.1 (6.8) [23]	55.0 (6.9) [142]	55.0 (12.3) [2]	53.0 (6.9) [26]
Mild	46.7 (8.8) [82]	51.2 (8.8) [70]	44.1 (7.0) [43]	47.7 (6.1) [97]
Moderate	43.2 (8.6) [111]	45.1 (8.0) [15]	40.8 (7.2) [121]	42.0 (7.0) [59]
Severe	38.1 (9.8) [32]	50.5 (–) [1]	38.1 (7.6) [31]	37.9 (5.8) [6]
Mental health component		*		*
None	41.2 (8.7) [23]	42.7 (7.5) [142]	39.8 (11.4) [2]	50.4 (8.9) [26]
Mild	41.0 (8.4) [82]	40.4 (7.9) [70]	45.0 (10.5) [43]	45.6 (8.8) [97]
Moderate	41.3 (8.3) [111]	41.2 (8.7) [15]	42.0 (10.2) [121]	42.5 (9.8) [59]
Severe	38.8 (8.9) [32]	50.5 (–) [1]	39.4 (13.3) [31]	31.7 (11.1) [6]

* *P* < 0.05; NR = not reported

## Abbreviations

ANOVA: Analysis of variance
B&B: Biberoglu and Behrman scale
BP: Bodily pain
CFA: Confirmatory factor analyses
CFI: Comparative Fit Index
CGI-C: Clinical global impression of change
CI: Confidence interval
*df*: Degrees of freedom
ES: Effect size
GH: General health
HRQOL: Health-related quality of life
MCS: Mental Component Summary Score
MH: Mental health
MID: Minimally important difference
PCS: Physical Component Summary Score
PF: Physical functioning
PROs: Patient-reported outcomes
RE: Role limitations due to emotional problems
RMSEA: Root mean square error of approximation
ROC: Receiver operating characteristic
RP: Role limitations due to physical problems
SEM: Standard error of measurement
SF: Social functioning
SF-36: Medical Outcomes Study Short Form 36
SRM: Standardized response mean
SRMR: Standardized root mean residual
VAS: Visual analog scale
VT: Vitality
